# Growth hormone promotes hepatic gluconeogenesis by enhancing BTG2–YY1 signaling pathway

**DOI:** 10.1038/s41598-021-98537-0

**Published:** 2021-09-23

**Authors:** Jeong-Rang Jo, Seungwon An, Swati Ghosh, Balachandar Nedumaran, Yong Deuk Kim

**Affiliations:** 1 Research Institute of Aging and Metabolism, Kyungpook National University, Daegu, 41566 Republic of Korea; 2 Department of Ophthalmology and Visual Sciences, University of Illinois at Chicago, Chicago, IL 60612 USA; 3 Department of Pediatrics, School of Medicine, University of Colorado Anschutz Medical Campus, Aurora, CO 80045 USA; 4 Barbara Davis Center for Diabetes, School of Medicine, University of Colorado Anschutz Medical Campus, Aurora, CO 80045 USA

**Keywords:** Biochemistry, Molecular biology, Physiology

## Abstract

Growth hormone (GH) is one of the critical factors in maintaining glucose metabolism. B-cell translocation gene 2 (BTG2) and yin yang 1 (YY1) are key regulators of diverse metabolic processes. In this study, we investigated the link between GH and BTG2–YY1 signaling pathway in glucose metabolism. GH treatment elevated the expression of hepatic *Btg2* and *Yy1* in primary mouse hepatocytes and mouse livers. Glucose production in primary mouse hepatocytes and serum blood glucose levels were increased during GH exposure. Overexpression of hepatic *Btg2* and *Yy1* induced key gluconeogenic enzymes phosphoenolpyruvate carboxykinase 1 (PCK1) and glucose-6 phosphatase (G6PC) as well as glucose production in primary mouse hepatocytes, whereas this phenomenon was markedly diminished by knockdown of *Btg2* and *Yy1*. Here, we identified the YY1-binding site on the *Pck1* and *G6pc* gene promoters using reporter assays and point mutation analysis. The regulation of hepatic gluconeogenic genes induced by GH treatment was clearly linked with YY1 recruitment on gluconeogenic gene promoters. Overall, this study demonstrates that BTG2 and YY1 are novel regulators of GH-dependent regulation of hepatic gluconeogenic genes and glucose production. BTG2 and YY1 may be crucial therapeutic targets to intervene in metabolic dysfunction in response to the GH-dependent signaling pathway.

## Introduction

Growth hormone (GH) is produced by somatotropic cells of the anterior pituitary gland. GH binding to GH receptor results in activation of Janus kinase 2 (Jak2) molecules and signal transducer and activator of transcription (STAT) family of transcription factors, which regulate expression of multiple target genes in response to GH signaling^1^. GH plays an important role in the regulation of aging, cancer, glucose metabolism, and lipid metabolism^1,2^. Both GH deficiency and GH excess are associated with disruptions in carbohydrate metabolism. Moreover, GH is closely linked to decreased oxidation and uptake of glucose in skeletal muscle and increased hepatic gluconeogenesis leading to insulin antagonist effects^2,3^. It is well-documented that GH is increased during fasting and suppressed during feeding state^4,5^. A recent report suggests that alterations in glucose metabolism and diabetes are common complications of acromegaly caused by excess production of GH^6^.

Glucose is used as a major energy source in organisms, from bacteria to humans. The liver stores the excess glucose as glycogen and supplies glucose during fasting. Hepatocytes produce glucose through gluconeogenesis under prolonged fasting condition^7^. Hepatic gluconeogenesis is generally associated with the transcriptional regulation of rate-limiting key enzymes, such as phosphoenolpyruvate carboxykinase 1 (PCK1) and glucose-6 phosphatase (G6PC). These hepatic gluconeogenic genes are regulated by numbers of transcription factors, nuclear receptors, and coregulators, including cAMP-response element-binding protein (CREB), forkhead box protein O1 (FOXO1), CCAAT/enhancing-binding protein alpha (C/EBPα), estrogen-related receptor gamma (ERRγ), peroxisome proliferators-activated receptor γ coativator-1α (PGC-1α), CREB-regulated transcription coactivator 2 (CRTC2)^8–14^. Especially, cAMP-PKA-CREB signaling pathway stimulates the expression of hepatic gluconeogenic genes and PGC-1α^8^. Hepatic deletion of FOXO1 markedly decreases both glycogenolysis and gluconeogenesis in mice^9^. C/EBPα is known to increase the gluconeogenesis^7,10^, whereas hepatocyte-specific deletion of C/EBPα is reported to significantly decrease gluconeogenesis^11^.

B-cell translocation gene 2 (BTG2) is an anti-proliferative gene and it is downregulated in many human cancers^15,16^. Previous reports showed that BTG2 is induced by growth factors in several cell types^17,18^. BTG2 is highly expressed in the liver and it can also be detected in various other tissues. Our previous study demonstrated that BTG2 acts as a crucial coactivator of CREB to regulate hepatic gluconeogenesis in hepatocytes^19^ and as a positive regulator of hepatic gluconeogenesis via the induction of Nur77 in diabetic mouse model^20^. Yin Yang 1 (YY1) is a member of the polycomb protein family and functions as a transcription factor. It is predominantly expressed in diverse tissues and involved in the regulation of multiple target genes via chromatin modification^21–23^. Lu et al. demonstrated that YY1 promotes gluconeogenesis through glucocorticoid receptor in the livers of mice^24^. It has also been shown that YY1 represses insulin/insulin-like growth factor (IGF)-signaling activation and skeletal muscle-specific YY1 knockout mice improved glucose tolerance and insulin-signaling activation^25^. However, the critical role of BTG2 and YY1 in regulating the GH-dependent hepatic glucose metabolism remains unexplored.

In this study, we demonstrated that GH treatment significantly increased hepatic gluconeogenesis via the induction of BTG2 and YY1 gene expression. Moreover, disruption of *Btg2* and *Yy1* markedly attenuated GH-mediated induction of hepatic gluconeogenesis. Our findings suggest that BTG2 and YY1 are key regulators of GH-induced hepatic gluconeogenesis. Therefore, BTG2 and YY1 may be novel potential therapeutic targets to combat metabolic dysfunction in response to the GH-dependent signaling pathway.

## Results

### Growth hormone increases hepatic BTG2 and YY1 gene expression

**Figure 1:**
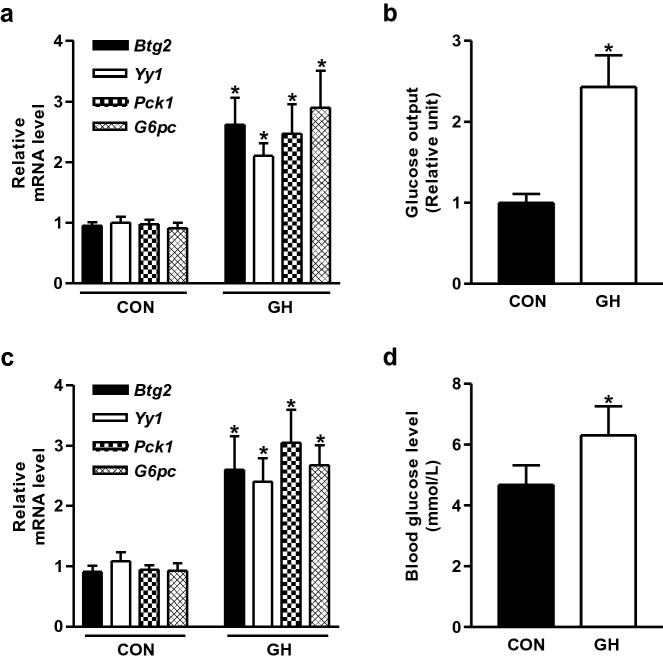
Growth hormone elevates hepatic gluconeogenesis in mouse primary hepatocytes and mouse liver. (**a**) Mouse primary hepatocytes (MPH) were treated with growth hormone (GH, 500 ng/ml) for 3 h. Gene expressions were analyzed by qPCR using the indicated primers. (**b**) Glucose output assay in MPH exposed to GH for 3 h. (**c**) Male C57BL/6 wild-type (WT) mice were injected intraperitoneally with GH (2 μg/g) for 7 days. (**d**) Blood glucose concentrations were measured from the indicated groups. n = 5 mice per group. *P < 0.05 vs. untreated control cells and mice.

Our previous report demonstrated that GH increased hepatic gluconeogenesis, but this phenomenon was abolished by metformin-ataxia telangiectasia mutated (ATM)-AMP-activated protein kinase (AMPK)-small heterodimer partner (SHP) signaling pathway^26^. Here, we examined more deeply the role of GH on hepatic gluconeogenesis in mouse livers and primary mouse hepatocytes. GH treatment significantly increased the levels of *Pck1* and *G6pc* along with the increased expression of *Btg2* and *Yy1* (Fig. 1a) in primary mouse hepatocytes. As expected, glucose production was efficiently elevated by GH treatment (Fig. 1b). Consistent with primary mouse hepatocytes data, GH challenge increased the mRNA levels of *Btg2*, *Yy1*, *Pck1*, and *G6pc* in mouse livers (Fig. 1c). Similarly, GH exposure significantly increased blood glucose levels relative to that of the control groups (Fig. 1d). Overall, these findings strongly suggest that GH plays an important role in hepatic gluconeogenesis and it can also induce *Btg2* and *Yy1* gene expression.

### Growth hormone-mediated induction of hepatic gluconeogenesis is BTG2 dependent

**Figure 2:**
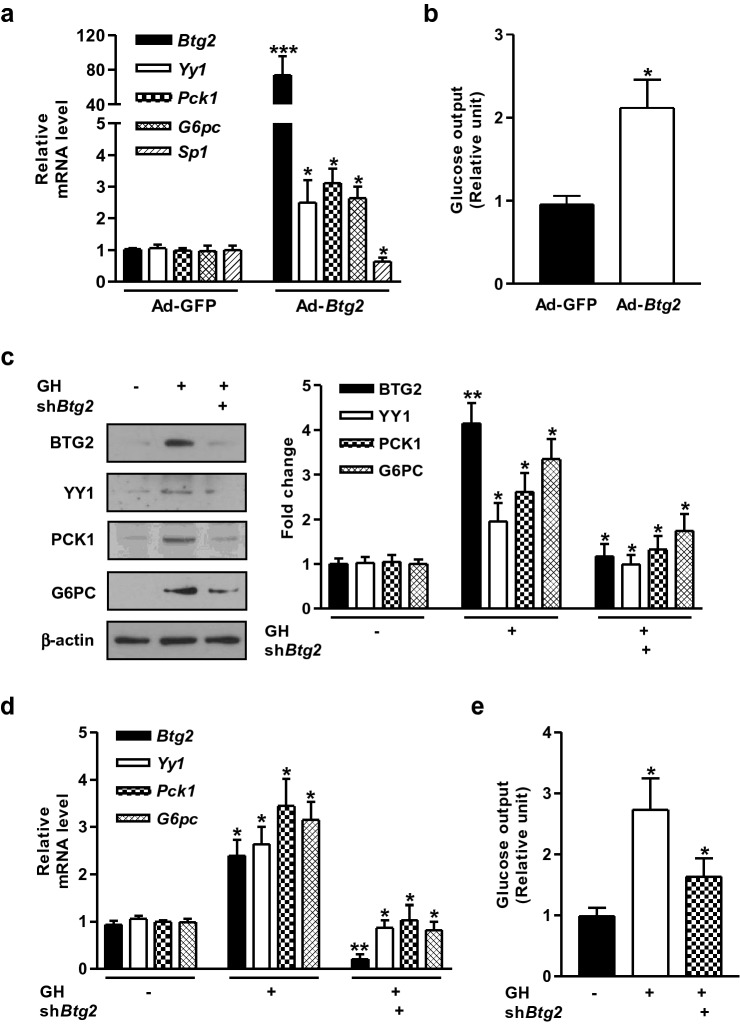
Induction of hepatic gluconeogenesis by GH is BTG2 dependent. (**a**) MPHs were infected with Ad-GFP and Ad-*Btg2* at a multiplicity of infection (MOI) of 60 for 36 h. qPCR analysis was performed to examine the expression of indicated genes using specific primers. *Sp1* is a negative control. (**b**) Glucose production assay in MPHs infected with Ad-GFP and Ad-*Btg2* for 36 h. (**c**) Whole cell extracts from MPHs of the indicated groups were subjected to immunoblot analysis with the indicated antibodies. (**d**) MPHs were infected with lentiviral-sh*Btg2* (sh*Btg2*) at 60 MOI for 36 h, followed by GH treatment for 3 h. Total RNAs were utilized for qPCR analysis with gene-specific primers. (**e**) Glucose production assay in MPHs infected with sh*Btg2* for 36 h, and then treated with GH for 3 h. *P < 0.05, **P < 0.01, and ***P < 0.001 vs. untreated control, Ad-GFP, or GH-treated cells.

Next, we investigated the critical role of BTG2 as a key modulator of gluconeogenesis using an adenoviral delivery system for the overexpression of *Btg2* (Ad-*Btg2*) or the control green fluorescent protein (Ad-GFP) in primary mouse hepatocytes. As shown in Fig. 2a, the *Btg2* mRNA was robustly increased by Ad-*Btg2*. Overexpression of *Btg2* significantly increased the expression of *Yy1*, *Pck1*, and *G6pc* compared to Ad-GFP control groups, but not the expression of specificity protein 1 (*Sp1*), a zinc finger transcription factor known to regulate many cellular processes. Interestingly, glucose production was also increased by Ad-*Btg2* compared to Ad-GFP control groups (Fig. 2b). Next, we further verified whether BTG2 modulates GH-mediated induction of gluconeogenic gene expression and glucose production in primary mouse hepatocytes. The GH-induced protein and mRNA levels of BTG2, YY1, PCK1, and G6PC were dramatically downregulated by endogenous knockdown of *Btg2* (Fig. 2c,d). Consistently, the increase of hepatic glucose production induced by GH treatment was markedly impaired when *Btg2* was silenced (Fig. 2e). Taken together, these findings imply the role of BTG2 in mediating GH-induced hepatic gluconeogenesis.

### Growth hormone- and BTG2-induced hepatic gluconeogenesis depends on YY1

**Figure 3:**
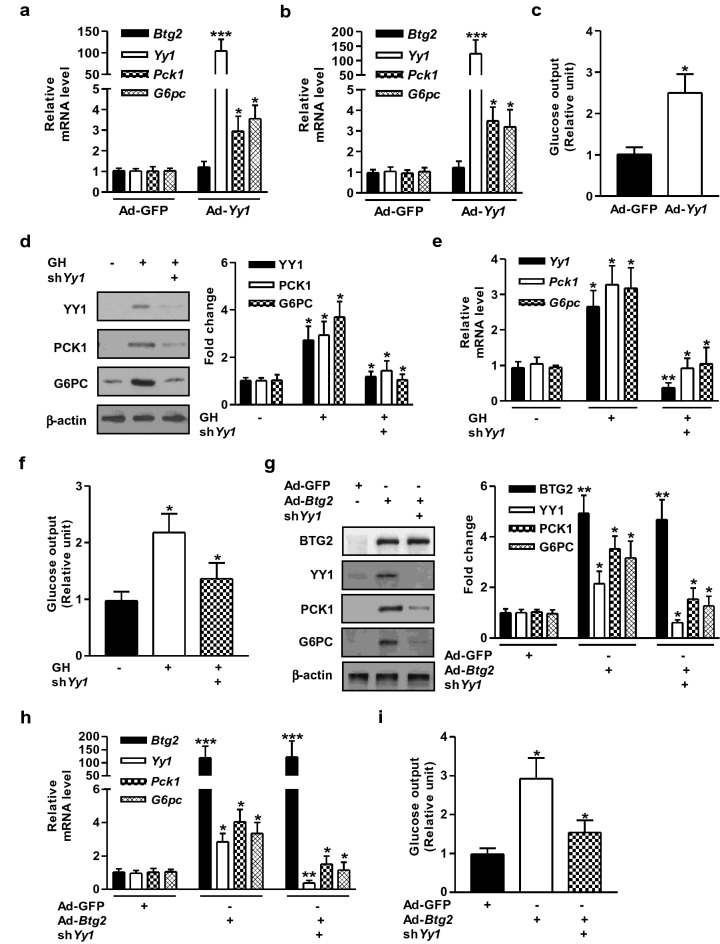
GH- and *Btg2*-stimulated hepatic gluconeogenesis is mediated by YY1. (**a**) MPHs were infected with Ad-GFP and Ad-*Yy1* at 60 MOI for 36 h. Gene expressions were analyzed by qPCR using gene-specific primers. (**b**) WT mice were injected with Ad-GFP and Ad-*Yy1* via tail-vein for 7 days. Indicated gene expressions were analyzed by qPCR with specific primers. (**c**) Glucose production assay in MPH infected with Ad-GFP and Ad-*Yy1* for 36 h. (**d**) MPHs were infected with lentiviral-sh*Yy1* (sh*Yy1*) at 60 MOI for 36 h and followed by GH for 3 h. Total protein was isolated and analyzed using Western blot analysis with various antibodies. (**e**) MPHs were infected with lentiviral-sh*Yy1* (sh*Yy1*) at 60 MOI for 36 h and then exposed with GH for 3 h. Total RNAs were utilized for qPCR analysis with indicated primers. (**f**) Glucose production assay in MPHs infected with sh*Yy1* for 36 h, and then treated with GH for 3 h. (**g**) MPH were infected with Ad-GFP, Ad-*Btg2*, and sh*Yy1* at 60 MOI for 36 h. Whole cell extracts were analyzed using Western blot analysis with various antibodies. (**h**) MPHs were infected with Ad-GFP, Ad-*Btg2*, and sh*Yy1* at 60 MOI for 36 h. Quantitative PCR analysis was performed using gene-specific primers. (**i**) Glucose production assay was performed in MPHs infected with Ad-GFP, Ad-*Yy1*, and sh*Yy1* for 36 h. *P < 0.05, **P < 0.01, and ***P < 0.001 vs. untreated control, Ad-GFP, GH-treated cells, or Ad-*Btg2*-infected cells.

To investigate the vital role of YY1 on hepatic gluconeogenesis by GH in primary mouse hepatocytes and the liver of mice. *Yy*1 expression was successfully overexpressed by adenoviral delivery system. Overexpression of Ad-*Yy1* significantly augmented *Pck1* and *G6pc* mRNA expression compared to Ad-GFP control groups in primary mouse hepatocytes and mouse livers, but not *Btg2* expression (Fig. 3a,b). Notably, glucose production was efficiently elevated by GH treatment (Fig. 3c). We further explored whether YY1 is involved in hepatic gluconeogenic gene regulation and glucose production. The protein and mRNA levels of YY1 were successfully silenced in GH-treated primary hepatocytes using lentiviral delivery system for shRNA *Yy1* (sh*Yy1*). The increase of gluconeogenic genes by GH treatment was markedly reduced in *Yy1* silenced group (Fig. 3d,e). Interestingly, GH-mediated induction of hepatic glucose production was also prominently decreased in *Yy1* knockdown group (Fig. 3f).

To determine whether BTG2-mediated induction of gluconeogenic key enzymes and glucose production can be modulated by YY1, we evaluated the effect of *Yy1* on the regulation of hepatic gluconeogenesis using Ad-*Btg2* and sh*Yy1* in primary mouse hepatocytes. Ad-*Btg2* significantly increased the expression of YY1, PCK1, and G6PC genes, while this phenomenon was markedly diminished by silencing of *Yy1* in primary hepatocytes, but not the expression of BTG2 (Fig. 3g,h). Similarly, Ad-*Btg2*-mediated increase in glucose production was markedly negated by silencing of *Yy1* (Fig. 3i). Overall, these results suggest that GH- and BTG2-stimulated hepatic gluconeogenesis is mediated by YY1.

### YY1 is a novel regulator of hepatic gluconeogenic gene transcription

**Figure 4:**
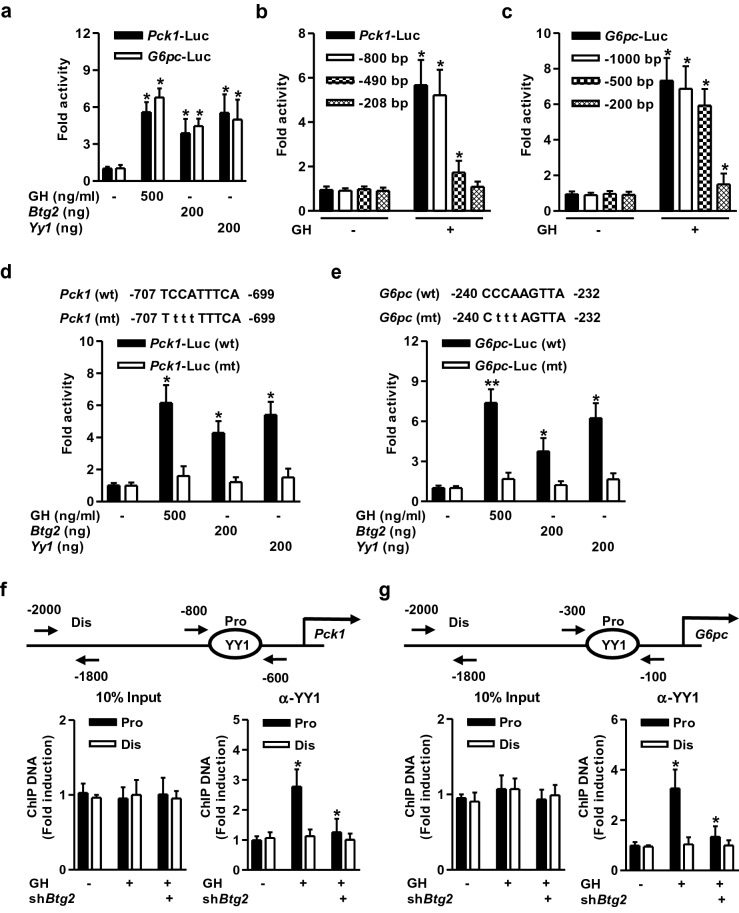
YY1 is a key regulator of hepatic gluconeogenic gene transcription. (**a**) AML-12 cells were transiently transfected with *Btg2*, *Yy1*, and the indicated reporter genes for 36 h. After transfection, the cells were treated with GH for 3 h. (**b**, **c**) AML-12 cells were transfected with wild-type and serial deletion forms of the *Pck1* (**b**) and *G6pc* (**c**) reporter constructs for 36 h and then treated with GH for 3 h. (**d**, **e**) AML-12 cells were cotransfected with wild-type (wt) and mutant (mt) forms of the *Pck1* (**d**) and *G6pc* (**e**) gene promoter, *Btg2*, and *Yy1* for 36 h. After transfection, the cells were treated with GH for 3 h. Luciferase activity was normalized to β-galactosidase activity in each well. *P < 0.05, **P < 0.01 vs. untreated control cells. (**f**, **g**) Chromatin immunoprecipitation (ChIP) assay for the recruitment of YY1 on the *Pck1* (**f**) and *G6pc* (**g**) gene promoter. MPHs were infected with sh*Btg2* for 36 h, and then treated with GH for 3 h. Cell lysates were immunoprecipitated with an anti-YY1 antibody. Purified DNA samples were used to perform PCR using primers binding to the specific proximal (Pro) and nonspecific distal (Dis) regions on the *Pck1* (**f**) and *G6pc* (**g**) gene promoter. 10% of the soluble chromatin was used as an input. *P < 0.05 vs. untreated control or GH-treated cells.

To determine whether the transcriptional activity of *Yy1* in response to GH treatment regulates hepatic gluconeogenic gene expression at the transcriptional level, we examined transient transfection assays using luciferase reporter constructs containing the *Pck1* and *G6pc* gene promoters in AML-12 cells. As shown in Fig. 4a, GH treatment significantly increased the activity of *Pck1* and *G6pc* gene promoters in hepatocytes. In addition, transiently expressed *Btg2* and *Yy1* significantly elevated the promoter activities of *Pck1* and *G6pc* as compared to the control groups. To identify the putative transcription activation site by GH on hepatic gluconeogenic gene promoter, serial deletion constructs of the *Pck1* and *G6pc* gene promoters were used for transient transfection and luciferase reporter gene assay. The activity of *Pck1* promoter induced by GH treatment was retained with deletion up to − 800 bp and this activation was completely lost around the − 490 bp construct indicating the presence of GH activation site between − 800 and − 490 bp of *Pck1* promoter (Fig. 4b). Moreover, *G6pc* promoter activity in response to GH exposure was retained with a deletion up to − 500 bp, and it was lost when used the minimal promoter (− 200 bp) construct (Fig. 4c). These observations indicate that the YY1-binding element required for the GH response is located within the region between − 800 and − 490 bp on the *Pck1* gene promoter and − 500 and − 200 bp on the *G6pc* gene promoter.

Our in-silico analysis predicted that there is YY1-binding site on *Pck1* and *G6pc* promoters. To further evaluate the functional significance of the YY1-binding region on the *Pck1* and G6pc gene promoter, site-directed mutagenesis was carried out on the *Pck1* and *G6pc* gene promoters. Wild-type (*Pck1* wt) and the mutant reporter plasmid (*Pck1* mt) were transiently transfected with *Btg2* and *Yy1* in hepatocytes. GH treatment as well as overexpression of *Btg2* and *Yy1* significantly increased *Yy1*-dependent activity of *Pck1* gene promoter, and this phenomenon was prominently abolished in *Yy1*-mutant *Pck1* promoter (Fig. 4d). Similarly, GH treatment or transiently expressed *Btg2* and *Yy1* efficiently enhanced *G6pc* gene promoter, and this increase was significantly hampered in the YY1-binding mutated (mt) *G6pc* promoter (Fig. 4e). Overall, these results suggest that the YY1-binding site can be sufficient to mediate the activation of *Pck1* and *G6pc* gene promoters in response to GH treatment. Finally, we conducted chromatin immunoprecipitation (ChIP) assays in primary mouse hepatocytes to identify the YY1-binding site on the *Pck1* and *G6pc* gene promoter. GH exposure strongly enhanced YY1 occupancy on the proximal region (Pro, − 800/− 600) of the *Pck1* promoter but not in the non-specific distal region (Dis, − 2000/− 1800) of the *Pck1* promoter (Fig. 4f). Moreover, we identified GH-induced YY1 recruitment on the proximal region (Pro, − 300/− 100) of the *G6pc* promoter but not in the distal region (Dis, − 2000/− 1800) of the *G6pc* promoter (Fig. 4g). Overall, these findings strongly suggest that YY1 is recruited to both *Pck1* and *G6pc* gene promoters to mediate GH-induced *Pck1* and *G6pc* gene transcription.

## Discussion

In this study, we have demonstrated that GH promotes hepatic gluconeogenesis via the induction of the BTG2–YY1 signaling pathway, and it was markedly abolished by sh*Btg2* and sh*Yy1* gene silencing. Consistent with gene expression data showing upregulation of hepatic gluconeogenic gene expression and glucose production was also significantly increased by the BTG2–YY1 signaling pathway in primary hepatocytes. Based on these findings, we suggest that the BTG2–YY1 signaling network exerts as a critical factor for the regulation of hepatic gluconeogenesis during the GH-dependent pathway.

Cui et al. have demonstrated that upregulation of multiple target genes (BTG2, c-FOS, and SOCS3) by GH treatment are mediated by specific mechanisms involving C/EBPβ and basic leucine zipper (bZIP) family transcription factors in adipocytes^27^. However, the potential link between GH and BTG2 in the regulation of hepatic gluconeogenesis has not been studied yet. Consistent with previous report, we found that GH exposure significantly increased the expression of BTG2 and YY1 genes, along with the increased key hepatic gluconeogenic gene (*Pck1* and *G6pc*) expression as well as glucose levels in mouse livers and primary hepatocytes (Fig. 1). Therefore, we speculate that BTG2 might be an important factor to regulate hepatic gluconeogenic gene expression and glucose production in response to GH signaling.

Our previous study has revealed that the induction of BTG2 by gluconeogenic signals (fasting state, forskolin, and glucagon treatment) positively regulates hepcidin gene expression and hepatic hepcidin production involved in iron metabolism by stimulating YY1 expression^23^. BTG2 also elevated hepatic gluconeogenesis via the induction of Nur77 in the livers of diabetic mice^20^. However, several other studies have demonstrated that hepatic gluconeogenesis is regulated by hepatocyte nuclear factor (HNF)-4α, HNF-6, and STAT5 during GH exposure^26,28,29^. Particularly, we investigated whether BTG2–YY1 signaling pathway may affect hepatic gluconeogenic gene expression and glucose production in response to GH treatment in primary mouse hepatocytes and mouse livers. As anticipated, GH significantly induced hepatic gluconeogenic gene expression, glucose production, and blood glucose levels (Fig. 1), whereas this stimulatory effect of GH was strikingly reduced in *Btg2* or *Yy1* silenced group (Figs. 2, 3). Our current study suggests that GH is a crucial regulator of hepatic gluconeogenesis by upregulating the BTG2–YY1 signaling network. Therefore, the identified BTG2–YY1 signaling pathway seems to be critical for hepatic GH-dependent gluconeogenic signaling.

YY1 is well-characterized to regulate hepcidin gene expression through BTG2–YY1 signaling network in mouse livers and primary hepatocytes^23^. In addition, BTG2 participates in the regulation of hepatic glucose metabolism through Nur77 and CREB induction both in vivo and in vitro^19,20^. Based on these findings, we proposed a novel molecular mechanism that BTG2–YY1 axis mediated hepatic gluconeogenic gene expression. As shown in Fig. 4, recruitment of BTG2 and YY1 on key gluconeogenic enzyme promoters were confirmed in hepatocytes. In response to GH treatment, endogenous YY1 was directly recruited to the YY1-binding site (proximal) region of both *Pck1* and *G6pc* gene promoters. Overall, our findings suggest a novel link between hepatic gluconeogenic gene transcription and BTG2–YY1 signaling pathway. However, we cannot rule out the possibility that one or the other unexplored mechanisms by unknown transcription factors or coregulators to regulates target gene transcription.

**Figure 5:**
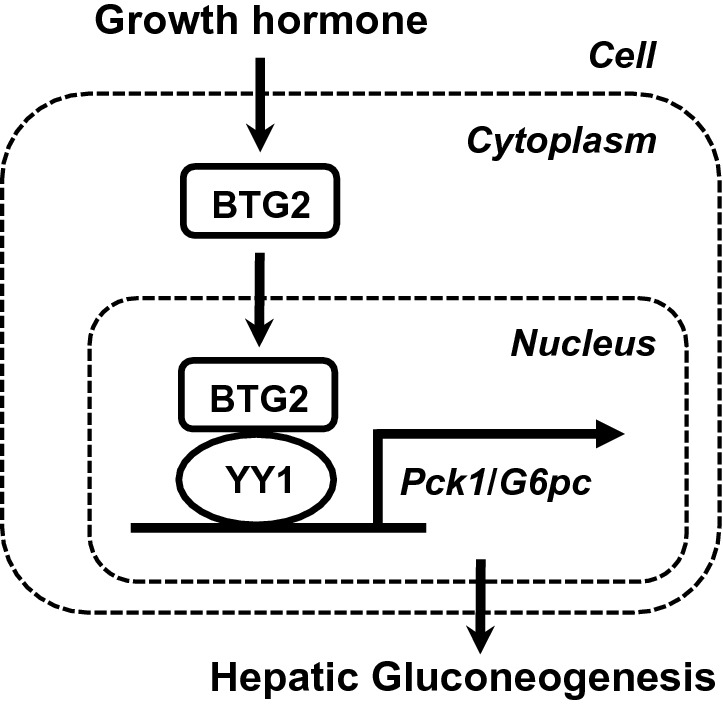
Proposed diagram for the regulation of hepatic gluconeogenesis by growth hormone. Growth hormone significantly increases hepatic gluconeogenesis via the upregulation of the BTG2–YY1 signaling pathway.

In conclusion, this present study demonstrates that GH augments hepatic gluconeogenesis by upregulating the BTG2–YY1 signaling pathway. Moreover, it suggests that BTG2–YY1 signaling pathway mediates GH-induced hepatic gluconeogenesis. Therefore, as described in Fig. 5, we propose a novel molecular mechanism involved in hepatic glucose metabolism by the BTG2–YY1 signaling network that may provide a better understanding to develop a novel therapeutic agent for metabolic dysfunction like diabetes.

## Materials and methods

### Animal experiments

8-week-old male C57BL/6 mice (Samtako, Osan, Republic of Korea) were used for the following experiments. For the growth hormone (GH) stimulation experiments, wild-type (WT) mice were injected intraperitoneally with GH (ProSpec, Tany Technogen, Ltd., Rehovot, Israel) (2 μg/g of body weight) for 7 days, as previously described^26^. At the end of the specified experiments, we euthanized the mice with CO_2_ and harvested liver tissues and blood samples. All animal experiments and protocols were approved and performed by the Institutional Animal Care and Use Committee of the Kyungpook National University according to the rules and procedures of the National Institutes of Health. The study was carried out following ARRIVE guidelines.

### Blood glucose level

Mouse blood was drawn from the tail vein and plasma glucose concentration was measured with ACCU-CHEK Active Glucometer (Roche Diagnostics, Mannheim, Germany) as reported^20^.

### Culture of primary mouse hepatocytes

Mouse primary hepatocytes (MPH) were isolated from the livers of 8-week-old male C57BL/6 mice (Samtako) by a two-step portal vein collagenase (Collagenase IA, Sigma-Aldrich, St. Louis, MO, USA) perfusion method and purified by centrifugation. Freshly prepared hepatocytes were cultured in M199 medium (Cellgro, Herndon, VA, USA), as described previously^20,23^. After trypan blue staining for determination of viability and attachment, cells were infected with the indicated adenoviruses, lentiviruses, and/or treated with GH (500 ng/ml).

### Cell culture and transient transfection assays

AML-12 cells (immortalized mouse hepatocyte) were cultured in DMEM/F-12 medium (Gibco-BRL, Grand Island, NY, USA) supplemented with 10% fetal bovine serum (FBS, Hyclone, Logan, UT, USA), insulin-transferrin-selenium (ITS, Gibco-BRL), dexamethasone (40 ng/ml, Sigma-Aldrich), and antibiotics (Gibco-BRL). The cells were maintained in a humidified atmosphere containing 5% CO_2_ at 37 °C. Transient transfection assays were performed with AML12 cells as reported previously^20,23^. Briefly, transient transfection was conducted using Lipofectamine 2000 (Invitrogen, Carlsbad, CA, USA) according to the manufacturer’s instructions. The total amount of DNA was adjusted to 0.8 μg per well by the addition of each appropriate amount of empty vector and β-galactosidase plasmids as the internal control. Luciferase activity was measured and normalized to β-galactosidase activity. All data are representative of at least three independent experiments.

### Construction of plasmids and DNA

The reporter plasmids encoding *G6pc*- and *Pck1*-Luc were kindly provided by Dr. Hueng-Sik Choi (Chonnam National University, Gwangju, Republic of Korea), as previously described^20^. Expression vectors of *Btg2* and *Yy1* were used as described previously^23^. The point mutation form of *G6pc* and *Pck1* promoter were generated using the site-directed mutagenesis kit (Stratagene, La Jolla, CA, USA) using the specific primers: *G6pc* forward, 5′-AGTGTGCTTTAGTTAATAAT-3′, and reverse, 5′-ATTATTAACTAAAGCACACT-3′ and *Pck1* forward, 5′-CATAGTTTTTTTTCAGGCAG-3′, and reverse, 5′-CTGCCTGAAAA-AAAACTATG-3′. All constructs or plasmids were confirmed by DNA sequencing.

### Glucose output assay

Glucose production from primary mouse hepatocytes was measured according to the manufacturer’s protocol using a colorimetric glucose oxidase assay kit (Sigma-Aldrich). Briefly, after plating, the cells were washed three times with phosphate-buffered saline (PBS), and then the medium was replaced with glucose production buffer (glucose-free DMEM, pH 7.4, containing 20 mM sodium lactate, 1 mM sodium pyruvate, and 15 mM HEPES without phenol red). The glucose production assays in the medium were conducted in triplicate as previously reported^19,20^.

### Recombinant adenoviruses

Adenoviruses encoding full-length of *Btg2* (Ad-*Btg2*), green fluorescent protein (GFP), and lentiviral delivery system of *Btg2*-targeted shRNA (sh*Btg2*) were described previously^23^. Ad-*Yy1* and recombinant lentiviral silencing system (sh*Yy1*) have been mentioned previously^30^. Adenoviruses were transduced at a multiplicity of infection (MOI) of 60 for 36 h in primary mouse hepatocytes, which was conducted according to the manufacturer’s instructions. Ad-*Yy1* (a single dose of 1 × 10^9^ plaque-forming units) were intravenously injected to WT mice for 7 days, as previously described^30^.

### RNA isolation and quantitative real-time PCR (qPCR) analysis

Total RNA was isolated from primary mouse hepatocytes and mouse livers using the TRIzol reagent (Invitrogen), and cDNA was synthesized using the Maxima^®^ First Strand cDNA synthesis kit (Fermentas, Vilnius, Lithuania), as previously mentioned^26,30^. Quantitative real-time PCR (qPCR) was performed using the SYBR Green PCR Master Mix (Applied Biosystems, Warrington, UK) and the StepOne™ Real-time PCR system (Applied Biosystem). We determine the expressions of *Btg2*, *Yy1*, *Pck1*, *Sp1*, and *G6pc* genes using qPCR, as previously described^20,23,31^. The expression of each target gene was normalized to ribosomal L32 expression.

### Western blot analysis

Protein lysates were isolated from liver tissues and primary mouse hepatocytes using a radioimmunoprecipitation assay (RIPA) lysis and extraction buffer (Elpis-Biotech, Dajeon, Republic of Korea). Proteins from whole-cell extracts were separated using 10% SDS-PAGE and transferred to nitrocellulose membranes (Amersham Biosciences, Piscataway, NJ, USA), as described previously^23,26^. The blotted membranes were probed with BTG2 (1:1000), YY1 (1:1000), PCK1 (1:1000), β-actin (1:3000, Santa Cruz Biotechnology, Santa Cruz, CA, USA), and/or G6PC (1:1000, Abcam, Cambridge, UK) antibodies. After incubation with the indicated antibodies, immunoreactive proteins were developed with an ECL Western Blot Detection Reagent (Amersham Biosciences).

### Chromatin immunoprecipitation assay (ChIP)

The ChIP assay was performed according to the manufacturer’s protocol, as described previously^23^. Briefly, primary mouse hepatocytes were fixed with 1% formaldehyde and sonicated. The soluble chromatin was subjected to immunoprecipitation using anti-YY1 (Santa Cruz Biotechnology) followed by incubation with protein A/G PLUS agarose bead (Santa Cruz Biotechnology) and salmon sperm DNA (Upstate Biotechnology, Lake Placid, NY, USA). After elution, DNA samples were recovered by phenol/chloroform extraction, and qPCR was performed using corresponding primers encompassing the proximal region of *G6pc/Pck1* promoter (*G6pc* forward 5′-CTCTGGCCTGGCTTCAAGGA-3′, reverse 5′-ACTTTTGTCTAAAATCTATT-3′, *Pck1* forward 5′-CTCTGGCCTGGCTTCAAGGA-3′, reverse 5′-ACTTTTGTCTAAAATCTATT-3′) and the non-specific distal region of *G6pc/Pck1* promoter (*G6pc* forward 5′-TCAATAATAACTGAGTTGAG-3′, reverse 5′-CGTCTGATATATCTCAAGTC-3′, *Pck1* forward 5′-TGCCATGGCTCACAGTGCCT-3′, reverse 5′-GTTACGAAATGACCTGGAGG-3′).

### Statistical analysis

Statistical analyses were performed using GraphPad Prism (GraphPad Software, CA, USA). Statistical significance of the differences between the two groups was determined using Student’s *t*-test, and multiple comparisons were analyzed using one-way analysis of variance (ANOVA). All data are expressed as mean ± standard error of mean. A *P* value less than 0.05 was considered statistically significant.
